# Understanding systemic factors in aging and rejuvenation

**DOI:** 10.18632/aging.104213

**Published:** 2020-11-13

**Authors:** Faisal J. Alibhai, Ren-Ke Li

**Affiliations:** 1 Toronto General Hospital Research Institute, Division of Cardiovascular Surgery, University Health Network, Toronto, Ontario, Canada; 2 Division of Cardiac Surgery, Peter Munk Cardiac Centre, Toronto General Hospital and University of Toronto, Toronto, Ontario, Canada

**Keywords:** aging, extracellular vesicles, senescence, rejuvenation, inflammation

Rejuvenation research is a rapidly expanding field aimed at restoring cell and tissue function of aged individuals. Pharmacological approaches include senolytic therapies which rejuvenate aged tissues via the removal and/or suppression of senescent cells [1]. Biological approaches provide cells/factors enriched in young individuals to restore aged tissue function. We previously demonstrated that transplanting young bone marrow stem cells into aged mice repopulates the aged bone marrow and restores repair responses of several tissues [2]. Others have shown that providing circulating factors though parabiosis of old and young mice improves tissue repair and stem cell function of old mice. Although several rejuvenation approaches have been shown to be effective, there remains a need to understand the factors and mechanisms which govern the pathophysiology of aging and beneficial effects of rejuvenation therapies.

Extracellular vesicles (EVs) are membrane bound vesicles which vary from nanometer to micrometer in size and carry a diverse set of factors (e.g. lipids, proteins, and microRNAs) that reflect the secreting cell’s milieu [3]. EVs are secreted by cells throughout the body and have emerged as key mediators of intercellular communication; however how aging affects their secretion and function is poorly understood. Recently, our group investigated how circulating EVs change with age, the cell types responsible, and the response of these factors to rejuvenation therapies. EVs were isolated from the plasma of young and old mice by size exclusion chromatography and the quantity, cargo, and function were assessed [4]. Quantification of EV markers revealed that EVs are more abundant in the old circulation whereas lipoprotein markers and total particles are lower. This suggests that aging affects multiple particle populations in the circulation. Examination of EV function revealed that old plasma-EVs reduced macrophage cytokine and polarization responses to LPS, reduced endothelial cell responses to VEGF, and increased macrophage phagocytosis, in a CD63+ particle dependent manner. Profiling of EV cargo revealed greater expression of inflammation-associated microRNAs such as miR-146a, miR-21, let-7a, and miR-223 in old plasma EVs compared with young. These microRNAs are predicted to target multiple intracellular signaling cascades which regulate cellular responses to external stimuli. To determine the cell type(s) responsible for changes in circulating EV microRNAs, we assessed EVs secreted by young and old peripheral blood mononuclear cells (PBMCs) *in-vitro* as well as plasma EVs isolated from old mice reconstituted with young or old bone marrow. However, EV microRNAs were similar in both models, suggesting that circulating cells have a minor contribution to the microRNAs identified this study. Further investigation into potential cell sources revealed that induction of senescence *in-vitro* and *in-vivo*, using gamma irradiation, mimicked the changes observed in old mice such as increased levels of circulating EVs and increased expression of EV associated miR-146a, mIR-21, and let-7a. Interestingly, senolytic therapy using dasatinib + quercetin (D+Q) reduced the expression of these microRNAs in the plasma of old mice, supporting that senescent cells or the pathways targeted by these compounds contribute to increased expression in the circulation. Collectively, this data demonstrates that aging and cellular senescence leads to increased levels of circulating EVs, and that these EVs impair cellular responses to activation. Pharmacological targeting of senescent cells partially rejuvenated the microRNA profile and functional effects of old plasma EVs.

Senescent cells have emerged as key players in the aging process. Senescent cells secrete cytokines, growth factors, and proteases which alter neighboring cell function. This secretome is collectively referred to as the senescent associated secretory profile (SASP) and the adverse effects of senescent cells are largely attributed to this profile. More recent definitions of the SASP have been expanded to include EVs [4,5]. During the induction of senescence EV secretion is substantially increased, potentially in response to a decline in the cell’s ability to recycle macromolecules. Secretion of EVs by senescent cells into the circulation could be one mechanism by which senescent cells promote cell dysfunction, as persistent uptake of senescent-EVs may lead to sustained changes in cellular function. Therefore, approaches aimed at targeting senescent cells may help reduce circulating senescent-EV levels and limit the impact senescent cells have on cells throughout the body. However, no surface markers specific for senescent-EVs have been reported, meaning selective identification and depletion of senescent-EVs from the circulation is currently not possible. Interestingly, other rejuvenation approaches such as exercise and activation of autophagy have been shown to affect EV section; modulation of EV secretion by these approaches may be one mechanism by which these therapies elicit beneficial effects.

Circulating factors that change with age can be divided into multiple categories but include factors enriched while young that decline with age, and aging-associated factors which are present at undetectable to low levels and increase [6]. The later play an active role in impairing cell function and should be considered in development of rejuvenation therapies. This notion is supported by a recent study which demonstrated that old blood rapidly impairs young cell function following a single blood exchange between young and old mice [7]. Interestingly, in a second study the rejuvenation actions of young blood were suggested to be in-part mediated by the dilution of old blood, further emphasizing the importance of aging-associated factors in rejuvenation [8]. Therefore, rejuvenation therapies may benefit from of a combination approach which provides young cells/factors and removes the aging-associated cells/factors. Future studies are needed to identify secreted factors which change with age, understand how these factors affect cell function, and determine the cell type(s) which change their secretory activity as we age.
